# Combining Fog Computing with Sensor Mote Machine Learning for Industrial IoT

**DOI:** 10.3390/s18051532

**Published:** 2018-05-12

**Authors:** Mehrzad Lavassani, Stefan Forsström, Ulf Jennehag, Tingting Zhang

**Affiliations:** Department of Information Systems and Technology, Mid Sweden University, 851 70 Sundsvall, Sweden; mehrzad.lavassani@miun.se (M.L.); stefan.forsstrom@miun.se (S.F.); ulf.jennehag@miun.se (U.J.)

**Keywords:** data mining, fog computing, IoT, online learning, monitoring

## Abstract

Digitalization is a global trend becoming ever more important to our connected and sustainable society. This trend also affects industry where the Industrial Internet of Things is an important part, and there is a need to conserve spectrum as well as energy when communicating data to a fog or cloud back-end system. In this paper we investigate the benefits of fog computing by proposing a novel distributed learning model on the sensor device and simulating the data stream in the fog, instead of transmitting all raw sensor values to the cloud back-end. To save energy and to communicate as few packets as possible, the updated parameters of the learned model at the sensor device are communicated in longer time intervals to a fog computing system. The proposed framework is implemented and tested in a real world testbed in order to make quantitative measurements and evaluate the system. Our results show that the proposed model can achieve a 98% decrease in the number of packets sent over the wireless link, and the fog node can still simulate the data stream with an acceptable accuracy of 97%. We also observe an end-to-end delay of 180 ms in our proposed three-layer framework. Hence, the framework shows that a combination of fog and cloud computing with a distributed data modeling at the sensor device for wireless sensor networks can be beneficial for Industrial Internet of Things applications.

## 1. Introduction

A current global trend is the digitalization of all aspects of our world. An example is applications that can utilize information from sensors attached to things in order to provide automatized operation and imitate intelligent behavior. This concept is commonly referred to as the Internet-of-Things (IoT) [1], and its application areas have spread to include industrial applications [2]. Most IoT solutions today are based on traditional client-server architectures with cloud back-ends, attached data analysis, visualization, and end user data access on the cloud side. This approach does, however, have some limitations, such as the cloud servers becoming single points of failure and an excessive delay when sending data to a cloud server far away on the Internet. A number of workarounds are used to avoid and address these limitations. One of the most recent workarounds is adding a fog layer to the cloud system [3]. When the fog servers act as cloud systems closer to the edges of the network, it can offload computationally heavy tasks of the IoT devices.

In the industry, wireless sensor networks (WSNs) have been a reality for some time, and research is being carried out on topics such as energy awareness, ad hoc routing, and reliable communication. WSNs are implemented through deployment of motes in usually harsh and/or hardly accessible environments. The sensor motes are mainly battery-powered and they are expected to remain active for a long period of time without human supervision and maintenance. Thus, it is preferable to expand a network’s lifetime by optimizing the energy consumption at mote level. Radio is the main source of energy consumption in motes, while idle listening, which requires as much energy as receiving, and overhearing are the main sources causing unnecessary draining of the battery. A common way to avoid unnecessary energy consumption is by using duty cycle management with Medium Access Control (MAC) protocols [4,5,6,7,8], where the battery life is extended by keeping the mote in sleep mode for most of the time. This solution, although effective for energy consumption, introduces a new problem: with a lower duty cycle the requirements of real-time applications cannot be met. Some research has proposed energy aware routing methods to solve this issue [4,9,10]. While recent solutions try to balance the trade-off between energy consumption and delay, the aim is to move away from duty cycle based MAC protocols towards wake-up radio systems [11]. Another challenge of WSNs concerns scalability. That is, how to reduce the communication for hundreds of sensor motes residing in the same network, and to better utilize the limited available bandwidth. This problem becomes more relevant in industrial scenarios. In coexistence with other wireless technologies such as 802.11 b/g/n/ac, WSN is prone to communication failure and packet loss, which results in retransmissions.

In recent years, many studies have been conducted to solve the aforementioned problems utilizing various methods of data mining and machine learning on large volumes of data generated by numerous motes in WSNs. However, while the availability of data in WSNs is promising for mining algorithms, the motes’ limited resources and volatility of generated data streams in WSNs pose great challenges for the traditional data mining algorithms. To overcome these challenges, many studies modified traditional data mining algorithms, to make them more suitable for deployment in WSNs, or suggested innovative approaches that consider the prerequisites of WSNs in the design phase [12]. There have been many important contributions in this field, but an important problem still exists: in literature, experiments are usually conducted with the assumption of access to a prior knowledge of the system, ground truth, and availability of data from all the resources (sensor devices) at any chosen time. These assumptions limit deployment of suggested models in real industrial environments, where sparsity of the collected data and dynamic system behavior are the norm.

**Figure 1:**
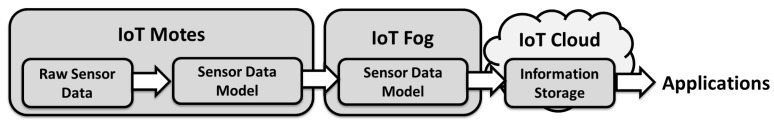
Proposed three layer framework for IIoT monitoring systems.

This article will explore how to solve some of the aforementioned problems. More specifically, how to save spectrum for the most urgent communications, and save energy in the motes by preventing unnecessary uplink transmission. We propose a distributed modeling method in a three-layer framework for Industrial IoT (IIoT) systems, consisting of a cloud back-end, a fog middle layer, and a lower WSN layer, see Figure 1. On the sensor layer we try to reduce energy consumption and unnecessary uplink transmissions. We achieve this by first creating a model on the sensor devices by mining the collected data that approximates the data stream behavior, and then transmitting only the updated model parameters to a fog computing system. To further enhance this, we will also utilize a distributed fog system to reduce the amount of data required to be sent to a large cloud system. We also investigate how introducing an IIoT can be beneficial to industrial monitoring systems. Hence, the research questions we aim to address in this work are:

1. To what extent can we save energy and reduce the number of uplink transmissions at the sensor motes, by introducing a model learned from data stream?
2. Can this model be practically implemented and utilized in a fog computing architecture?
3. What are the expected end-to-end delay and information query times of such a system, and can it be used in industrial scenarios?

The remainder of this article is structured as follows: Section 2 presents an overview of our approach and related work. Section 3 gives the details of our approach, including modeling the data stream at sensor devices and the distributed modeling process in the fog. Section 4 describes the details of the implemented testbed system. Section 5 presents the used hardware and measurement methods. Section 6 presents the results and evaluation of the proposed model, and the resulting system and their implications. Finally, Section 7 presents the conclusions and future work.

## 2. Data Mining and Machine Learning in WSNs

Tools and algorithms in data mining and machine learning have been used in many recent studies to create predictive models in sensor networks [13]. Kernel linear regression was used in an in-network approach where the sensor network tries to optimally fit a global function to each of the sensors’ local measurements [14]. The learned linear model takes the form of a weighted sum of local basis functions given by users, which provides an expressive yet tractable class of models for sensor network data.

Extended Kalman filters (EKFs) [15] have also been widely used in related works to predict the sensor value. To be able to use Kalman filters, the user needs to provide the next state function, the measurement function and noise term. In [16], a family of algorithms for training the noise parameters of an EKF in a sensor were proposed. The algorithms adjust the covariances of the EKF in a way that maximizes their prediction accuracy. To make use of the spatial information of the sensor nodes, distributed Kalman filtering estimates the system state based on the information not only from itself but also from its neighboring sensors according to the networks topology. In [17], an event-based distributed filter has been proposed in wireless sensor networks to reduce the sensor data transmission rate and the energy consumption. Each node makes decisions independently about when local messages need to be broadcast. While the presented results are promising, the paper does not provide any suggestions for further communications with cloud or fog nodes. Hence, filtering is only used to reduce number of transmissions and no mechanism is suggested to simulate the data stream in the central monitoring unit.

Dividing the sequences into meaningful subsequences and representing them by using real-valued functions is one of the most common and fundamental components of time series data mining [18]. In [19], trend change is detected by using Bayesian inference; the patterns are learned offline; finding the most accurate trend changing point is an optimization problem. In [20], evolutionary computation was suggested for time series segmentation of stock price data. A series segmentation was proposed by [21], which is achieved by a polynomial approximation of the time series using a basis of orthogonal polynomials. These methods require repetition and a relatively large storage, which makes deployment in WSNs infeasible.

A hierarchical three-layer deployment for energy efficient IIoT architecture is proposed in [22]. The high level architecture consists of a cloud server, a Representational State Transfer (REST) service, as well as sensing entities. The study mainly focuses on the sensing entities to efficiently reduce the energy consumption by optimizing their sensing, processing and communications. For this purpose, a three-layer framework is proposed, which divides nodes by functionality and extends network life by balancing traffic load and predicting sleep intervals. Although the framework in [22] carries similarities with our proposed framework, in terms of architecture and energy efficiency, the methodological aspect differs vastly. In [22], semi-centralized smart scheduling is deployed to achieve energy efficiency, whereas our approach utilizes a distributed adaptive data-driven modeling and event-based communication at the sensor motes to achieve the same goal.

Some recent articles focus on the communication and distribution problems that appear between sensor and fog devices. Li et al. [23] highlights scheduling problems in deep learning IoT edge devices, including performance evaluation of their proposed scheduling. Dautov et al. [24] also highlights distribution and communication problems when performing stream analytics on edge devices. They also investigate the differences of having traditional vertical device and a cloud structure, compared to a more horizontal device, fog, and cloud system. To the best of our knowledge there has so far not been any studies deploying a distributed data model utilizing fog computing for monitoring systems in IIoT.

### Approach

We aim to create a three layer framework for an industrial monitoring system to investigate and answer the previously presented research questions. An overview of the approach is found in Figure 1. The bottom layer consist of resource constraint sensor devices collecting field data, and the top layer is a cloud back-end with a view over the whole system. The fog layer connects the distributed view of the WSN layer to the overall view of the cloud layer. At the lower layer, a sensor device starts by learning parameters that describe the data stream in the best way to create an initial model. Then it continues to monitor the data stream and update the model parameters. When the sensor device detects a trend change or a necessary model parameter update, the new information will be sent to the associated fog node. The fog node will simulate the data streams based on the model with the received updated parameters. Through collection of updated model parameters from the associated sensor devices, a synthesis directed probability graph can be created in the fog node. This graph provides the fog node with a view of the operational state on the associated monitoring area, making it possible to detect local anomalies, trends and sensor devices’ faulty behavior. The global model of the directed graph is constructed in the cloud by collecting all local graphs from the fog nodes. Anomalies, changes in trend and faults in the system can be detected in the cloud by monitoring the parameter changes in the global graph.

In the next section we present the data streams learning and monitoring model for the sensor network layer and fog computing layer. With respect to the research questions stated in Section 1, the construction of the global graph in the cloud computing layer, and a more sophisticated fault detection system are out of the scope of the current study, and we leave these tasks for future work.

## 3. Proposed Data Streams Learning and Monitoring Model

**Figure 2:**
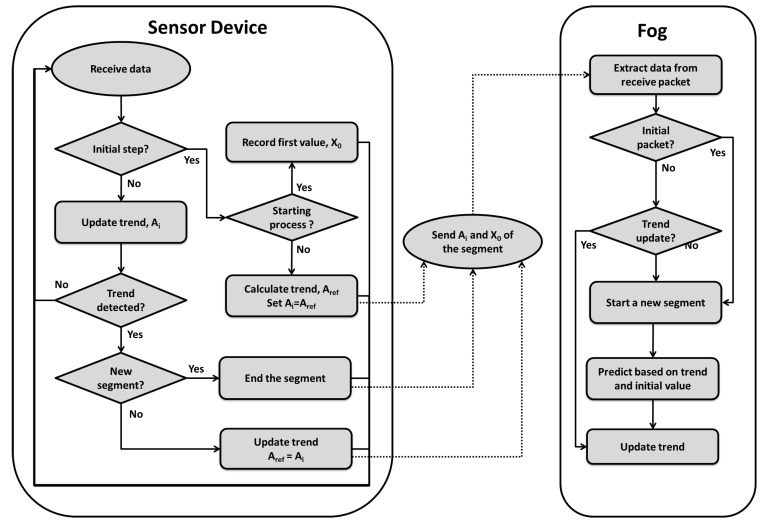
The learning process in sensor devices and simulation process in fog node.

This approach aims to reduce the communication between sensor motes and fog nodes to save energy and spectrum. Therefore, we divide the problem into learning and monitoring in the sensor device, and simulation of the data streams in the fog node. A sensor device learns an initial model from the data streams and when it detects a trend change or a necessary model parameter update, the new information will be sent to the associated fog node. The fog node will simulate the data streams based on the model with the received updated parameters. In the sensor motes residing in the WSN layer, we aim to utilize a novel energy saving and packet reducing technique, to address the first research problem. This will be done in order to make the motes last longer on battery and to send as little data as possible to open up for higher priority data transmission. Instead of sending a large number of small data packets, the sensor devices will calculate parameters of a mathematical model from the raw values and send the updated parameters of the model to the fog. This means sending new data only when changes are detected and the model needs to be updated. In the fog layer an approximation of the original data stream is simulated by using the updated model parameters for a duration of one segment, which is the interval between the two last packets, received from sensor devices in the WSN layer. Furthermore, the information from the fog layer is sent to the cloud to provide a view of the whole system and other services required by end users. A flow chart of the learning process in the sensor device and the simulation process in the fog node can be seen in Figure 2.

### 3.1. Learning and Monitoring in the Sensor Device

The operation of a sensor device consists of two phases, which can also been seen in the flow chart. Namely: The initialization phase, and the monitoring phase.

#### 3.1.1. Initialization Phase

In the initialization phase, the sensor device collects data stream values for a certain number of data points *m*, the maximum segment length, to set the parameters of an initial linear regression model. In this phase we assume that the system behavior is normal, that there are no anomalies or errors in the collected data. A sensor device normalizes the collected values and calculates a reference value for a set of statistical information, ζ, which will be required to detect changes in the data stream and for the fog node to simulate the segments of the data stream. For each segment, the sensor device extracts the following statistical information.

The reference slope

(1) $$A_{ref}=\left(x_{m-1}-x_{0}\right)/m;$$

The sum of the values:

(2) $$sumX=\sum_{i=0}^{m-1}x_{i};$$

The second moment (surprise number [18]):

(3) $$surpriseX=\sum_{i=0}^{m-1}x_{i}^{2};$$

And the step calculated as:

(4) $$stepX=\sum_{i=0}^{m-1}ix_{i}.$$

By the end of the initial phase, the sensor device, sends the initial model parameters, $A_{ref}$, the first value of the first segment $x_{ref}$ and starting time point $t_{0}$ to the fog node. The process of the sensor device in the initialization phase is presented in Algorithm 1.
 **Algorithm 1:** Initialization Phase. 
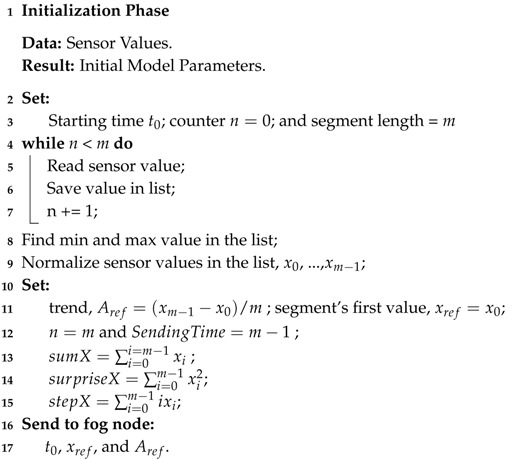



#### 3.1.2. Monitoring Phase

In this phase a sensor device learns the dynamics of the data stream, updates the model parameters, detects changes, and communicates this information to the fog node. Algorithm 2 represents the process of the monitoring phase. The sensor device carries out the task by reading the data stream one time point at the time, for maximum of *m* time points, which is the maximum length of each segment. The maximum length of each segment is the time interval between two consecutive packet transmissions. This is to overcome the issue of limited memory on the device, and to meet the requirements of minimum number of required communications in WSNs. At each time point, the sensor device reads the sensor value, updates the statistical information and predicts the behavior of the data stream in the next time point. When reading the next sensor value, the device calculates the root square error (RSE) of its prediction. If the prediction error is negligible and does not reach the specific prediction error threshold θ, the data stream shows a stable behavior, meaning that no change in trend has been detected. The sensor device then updates the model parameters and transmits a packet at the end of the current segment consisting of the statistical information $\zeta_{i}\left(A_{i},x_{0_{i}},n_{i},sumX_{i},surpriseX_{i},stepX_{i}\right)$ to the fog node. When the prediction error for any time point is larger than θ, it is an indication of a possible change in system behavior. The sensor distinguishes a trend from a state change, by checking the error value of the predicted trend for the current segment, and the error value of the predicted trend with respect to the reference trend, $A_{ref}$. If the evidence indicates that a trend has occurred, the sensor device updates the model parameters and transmits them in the scheduled transmission, at the end of the current segment. In cases where the detected change indicates a new state, the sensor device ends the current segment, transmits ζ to the fog node and starts the initialization phase to identify the new model parameters.
 **Algorithm 2:** Monitoring Phase. 
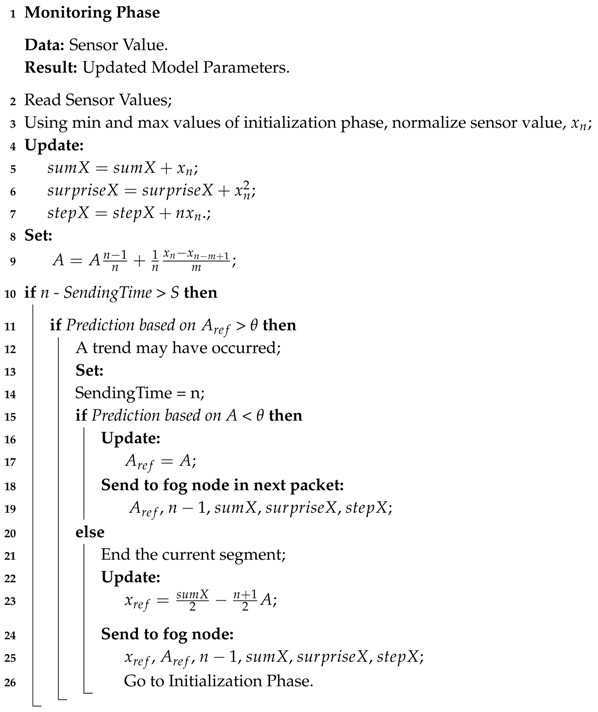



Having given an overview of the proposed learning and monitoring model, we formulate the model of the sensor device. Let $X=\{x_{0},x_{1},x_{2}\ldots ,x_{T}\}$ be a data stream sampled at uniform and ordered time points t=0,…,T. Then any time point *k*, 0<k<t, of a polynomial temporally correlated data stream, with respect to a starting point $t_{0}$, can be represented as:

(5) $$f\left(t\right)=\sum_{i=0}^{k}a_{i}\left(t_{i}-t_{0}\right)+d\left(t_{0}\right)+\epsilon ;$$

where $a_{i}$ and *d* are constant coefficients describing the characteristics of the stream, slope and intercept respectively, and ϵ is a small random value with standard normal distribution. According to the assumption of uniform and ordered time points we have $t_{1}-t_{0}$ = $t_{i}-t_{i-1}$, for i=1,…,n. We also know that according to Equation (5) for any segment with length l=1,…,k and k<n:

$$f_{0}\left(t_{n}\right)=f\left(t_{n}\right),$$

(6) $$f_{l}\left(t_{n}\right)=f_{l-1}\left(t_{n}\right)-f_{l-1}\left(t_{n-1}\right).$$

If $a_{i}\left(t_{i}-t_{0}\right)=A_{ref}$, where $A_{ref}$ is the reference value of the slope (trend), to model the data stream behavior and estimate the next step, it is reasonable to assume that in the absence of a trend change in a data stream, Equation (5) for the *n*th time point, can be simplified to:

(7) $$f\left(t_{n}\right)=A_{ref}+\epsilon ,$$

and, prediction of the next time point, n+1, for any chosen point in the data stream segment, $f_{k}$, can be approximated by:

$$f_{k}\left(t_{n+1}\right)\approx A_{ref}.$$

So the next value of the *l*th segment can be approximated as:

(8) $$f_{l}\left(t_{n+1}\right)\approx f_{l-1}\left(t_{n+1}\right)+f_{l-1}\left(t_{n}\right).$$

It should be mentioned that we do not impose any restriction on the interval value between two consecutive time points, as long as the intervals are the same for all the points in the data stream. Indeed, in Section 6, we show that our proposed model performs well on various data streams with different sampling rates. Another task of a sensor device is to predict the trend for each segment. For a segment $f_{l}\left(t_{n}\right)$ in any given data stream, a trend can be calculated as:

(9) $$A_{l}=E\left[f_{l}\left(t_{n}\right)\right];$$

where E[.] is the average function, and $A_{l}$ is the segment’s trend. The problem arises when considering the existing random noise in collected sensor values, which poses negative effect on the approximation accuracy. This negative effect makes it more difficult to predict trends or detect new states, especially when the standard deviation of a data stream, $VAR(f\left(t_{n}\right))=\sigma$), is a large value, or a value close to the prediction error threshold θ. However, since the random noise is independent, the standard deviation function can be rewritten as:

(10) $$VAR(\sum_{i=1}^{m}\left(f_{l}\left(t_{n-i+1}\right)\right)=\sqrt{m}\;\left(VAR\left(f_{l}\left(t_{n}\right)\right)\right)=\sqrt{m}\;\sigma .$$

Hence, we can rewrite the estimation of trend as:

(11) $$A_{l}=E\left[\frac{\sum_{i=1}^{m}f_{l}\left(t_{n-i+1}\right)}{m}\right],$$

with $\frac{\sigma }{\sqrt{m}}$ as the standard error of the estimation.

At the end of each segment the sensor device sends statistical information of the normalized segment, $f_{l}\left(t_{i}\right)$, to the fog node. As mentioned previously, one of the main challenges for a sensor device is the limited resources, namely memory and processing. To overcome this problem, a stepwise process updates the model parameters. In other words, instead of iterating over all values in the segment to update the required parameters, a sensor keeps one value for each of the pieces of statistical information of a segment and updates the values as follows:

$$sumX=sumX+x_{n},$$

$$surpriseX=surpriseX+x_{n}^{2},$$

$$stepX=stepX+nx_{n}.$$

### 3.2. Simulation in the Fog Node

The main task of a fog node is to collect the summarized information from the associated sensor devices, and further merge these small segments into a longer trend segment and different states. The complex system of multi-nonlinear data streams can be simply described as a succession of sequential multi-linear streams, which can be described by a probability graph network model. The model is defined as a directed graph G=(V,E), where $V=s_{i}$ is a set of vertices or nodes with state $s_{i}$. Each state $s_{i}$ , represents multiple data streams in which the temporal correlation of each of the streams has not changed in a specific time interval. A state can be defined as:

(12) $$s_{i}=\left\{{\vec{D}}_{i,j}\left(T\right)|\;\forall j\in \left\{i_{1},\ldots ,i_{k}\right\}:{\vec{D}}_{i,k}\left(\left[t_{s},t_{e}\right]\right)\in FD_{i,j};T\in \left[t_{start},t_{end}\right]\right\},$$

where ${\vec{D}}_{i,j}\left(T\right)$ is a finite subset of the data stream ${\vec{D}}_{i,j}$ for a time interval *T* ($t_{start}<t_{end}$), and $FD_{i,j}$ is a linear regression model that describes one sub data stream. An edge of the graph, $E=\{<s_{i},s_{j},p_{ij}>\}$, is a set of links that represent a switch from one node to another with some probability $p_{ij}$:

(13) $$p_{ij}=P\left(\vec{D}\left[t_{3},t_{4}\right]\in s_{j}\;|\;\vec{D}\left[t_{1},t_{2}\right]\in s_{i}\;\wedge \;\forall \;t_{3}:t_{3}-t_{2}>\delta \;\wedge \;\vec{D}\left[t_{1},t_{3}\right]\notin s_{j}\right).$$

Each state, $s_{i}$ , provides summary information about the nature of a subset of multiple data streams. After receiving statistical information from several sensor devices, a fog node constructs the graph by merging several short consecutive segments with the same trend, and adding a new node to the graph, that is if the node does not exist in the graph. If the node already exists, the fog node only updates the summary information of the state. To distinguish between a new state and an already existing state, a fog node compares the mean square error of the new trend $A_{i}$ with an acceptable error threshold δ. Given the summary information of the segment $\zeta_{i}\left(A_{i},x_{0_{i}},n_{i},sumX_{i},surpriseX_{i},stepX_{i}\right)$, the difference will be:

(14) $$\sqrt{\frac{surpriseX}{n_{i}}-2x_{0_{i}}\frac{sumX}{n_{i}}-\frac{2A_{i}}{n_{i}}stepX+x_{0_{i}}^{2}+\left(n_{i}+1\right)A_{i}+A_{i}^{2}\left(\frac{ni^{2}}{3}+\frac{n_{i}}{2}+\frac{1}{6}\right)}\;,$$

where $A_{i}$ is calculated as:

(15) $$A_{i}=\frac{\frac{2}{n_{i}}stepX-i\left(n_{i}+1\right)}{2(\frac{n_{i}^{2}}{3}+\frac{n_{i}}{2}+\frac{1}{6})}\;.$$

So the segments $i_{start}$ to $i_{end}$ can be merged if the following condition is true:

(16) $$\sqrt{\frac{surpriseX}{n_{i}}-2x0\frac{sumX}{n_{i}}+x_{0_{i}}^{2}-\frac{{\left(\frac{2}{n_{i}}stepX-\left(n_{i}+1\right)\right)}^{2}}{4(\frac{n_{i}^{2}}{3}+\frac{n_{i}}{2}+\frac{1}{6})}}\;<\;\delta .$$

Although the process of the cloud node is out of the scope of this work, it is easily conceivable that a process similar to the fog node can be carried out in the cloud to construct the meta graph and provide a complete overview of the system.

## 4. The Testbed System

**Figure 3:**
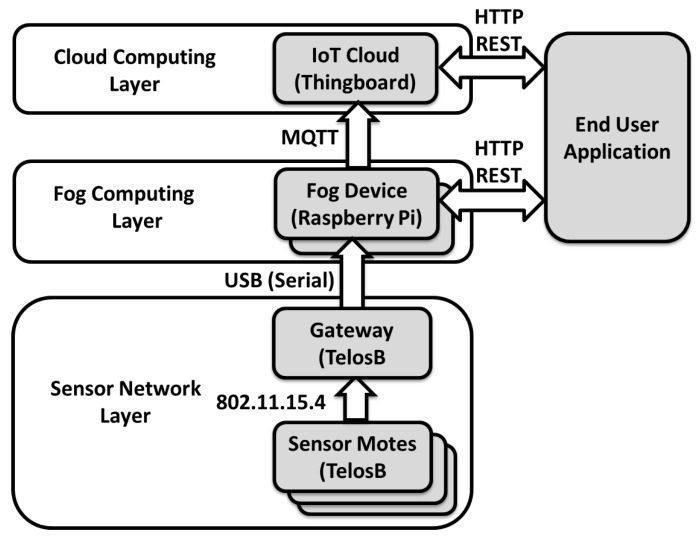
The testbed system consisting of cloud server, Raspberry Pi fog nodes, and TelosB sensor motes.

We have chosen to implement and test our approach as a real world implementation in a testbed system. Figure 3 shows an overview of this testbed system. The testbed system is split into three layers: the sensor network layer, the fog computing layer, and the cloud computing layer.

The sensor layer at the bottom is a wireless sensor network consisting of motes with both sensor devices and a gateway. It is in the sensor devices that the raw sensor data is collected for the proposed system, but these sensor devices could have very limited resources and power. The raw sensor values are made into the sensor data model by the sensor device, in accordance with the algorithm explained in Section 3. The model and updates to the model are then sent over IEEE 802.11.15.4 industrial wireless communication to a gateway node connected via USB to a fog computing device in order to push the information further up the system. The second layer is the fog computing layer, which has been implemented using small resource constrained computers. The purpose of the fog computing layer is to reduce the amount of data sent to the cloud, provide close to edge computational capabilities, and reduce the communication delay to end users, since they do not have to go through the cloud to get to the sensor values. The data model is received from the gateway mote, from which sensor values are generated and pushed upwards to the cloud at regular intervals. The final and top layer is the cloud computing layer. This cloud serves as a persistent storage point with scalable storage and computational power. This layer was implemented by using an IoT cloud platform to store the data in a database and display the sensor values in a more user-friendly manners.

### 4.1. Sensor Network Layer

Two types of motes are programmed: gateway and sensor. The gateway works as a connection between the sensor network layer and the fog computing layer; hence its role is limited to collecting data from the payload of packets received from sensors. The sensor motes are programmed to imitate the algorithm presented in Section 3. Correspondingly, sensor devices’ functionality is divided into three states, implemented utilizing the multi-threading module of Contiki [25] to switch between threads, according to the sensor device’s current state in the algorithm. Upon joining the network, sensor devices continuously collect sensor values, such as temperature and illumination, and save them in an internal flash memory for a user-defined sampling duration, $w_{init}$. The sensor device sends the first unicast message to the gateway when the sampling duration ends. The payload contains the minimum ($min_{init}$) and maximum ($max_{init}$) values observed during this time. When the sampling process is complete, the sensor device maintains a fixed length list, containing the most recent collected sensor values, in an internal flash memory. Motes are programmed to sample data periodically. The sampling frequency is set to one sample per second, which is a common choice for industrial monitoring systems. Each sampling event triggers a chain of mote-level calculations and evaluations to update the pre-defined relevant model parameters, explained in Section 3, and the change point detection. Unicast messages are sent to the gateway with updated values of model parameters, either if the current prediction error exceeds the hard-coded error threshold δ, or a sudden change within the accepted error range is detected (that is, the error has not exceeded δ value, but the change is significant enough to indicate a new period.)

### 4.2. Fog Computing Layer

The fog computing layer was implemented using small resource-constrained computers, namely Raspberry Pi devices. The purpose of the fog device is to interpret the model from the sensor motes, generate sensor values to send to the cloud, and act as a direct access point for the sensor values. A Java program was written to run on the fog devices for this purpose. To be specific, the program is split into four parts, which are implemented as concurrent threads. The sensor mote reader thread, the model interpreter thread, the cloud publisher thread and the REST interface thread. The sensor mote reader part reads from the gateway mote, which is connected via USB to the Raspberry Pi. The gateway mote created a virtual serial port, which is then used to communicate between the fog and gateway. When the gateway mote receives new data from the sensor mote, it sends the data model parameters to the gateway to be further interpreted. These model parameters include a device ID and the values for surpriseX, sumX, stepX, Xmin, Xmax, $x_{i_{0}}$ and $A_{i}$. The model interpreter uses these values to generate sensor values from the normalized model values using the formula Y=Xmin+x(Xmax−Xmin) in accordance with the established model parameters. This in turn is handled by the cloud publisher, which publishes sensor values to the cloud via the MQTT protocol on a fixed interval set to every two seconds. Finally, the REST interface is a thread that listens for an incoming HTTP GET connection on port 9999 in order to return the latest sensor value as a JSON object from the model in a HTTP response.

### 4.3. Cloud Computing Layer

The top layer is the cloud computing layer, which is implemented using the ThingsBoard IoT cloud platform running on a desktop computer as a server system. The purpose of this layer is to act as persistence storage of sensor values in a database and to show the sensor values in more user-friendly graphs and tables. The ThingsBoard IoT cloud system has many of these functions built in, including an MQTT broker and an MQTT client to listen and handle MQTT messages from the fog devices. Hence, the cloud system simply acts as a back-end storage system and sensor value visualizer, based on the sensor values sent from the fog layer.

## 5. Materials and Methods

This section will give a detailed description of the hardware and software used to implement the system as a testbed. It will also explain the details of setting up the measurements inside the testbed system.

### 5.1. Hardware and Software Materials

The hardware we used in the wireless sensor network layer was TelosB [26] motes with an IEEE 802.15.4 complaint transceiver, CC2420 [27] and CSMA/CA medium access control protocol, running the Contiki [25] operating system. The fog computing layer was created using Raspberry Pi model B+ hardware, running the Raspbian operating system version 9. The program running on the Raspberry Pi was written in Java 7, and when the getaway mote was connected to the Raspberry Pi, it created a virtual serial port at 115,200 baudrate, which is used by the Java program to communicate between the gateway mote and the Raspberry Pi. The Raspberry Pi was then connected to a local gigabit Ethernet network created by a Linksys WRT1200AC network router. The cloud computing layer was realized using regular desktop computer hardware, also connected to the Linksys WRT1200AC router. Hence, a Dell Optiplex 780 with a 2.93 GHz Intel Core 2 Duo processor and 4 GB of RAM was used as the cloud machine, running Ubuntu version 16.04 LTS and ThingsBoard 1.3.1. A laptop was also used for some of the measurements. This laptop was a HP Elitebook 1040 G3, with an Intel I7 2.5 GHz processor and 8 GB RAM, connected to the same Ethernet network provided by the Linksys WRT1200AC router.

### 5.2. Evaluation Methods and Setup

To verify that our testbed system and model are working as expected, a series of measurements and evaluations were performed on the system. Specifically, we decided to measure and evaluate the reduction in the number of packet transmission attempts by sensor devices due to implementation of the proposed model, the end-to-end delay of the proposed framework, the query times of fog and cloud, the scalability of fog, and finally the cost imposed on the sensor devices’ awake time in terms of computation of model parameters.

**Figure 4:**
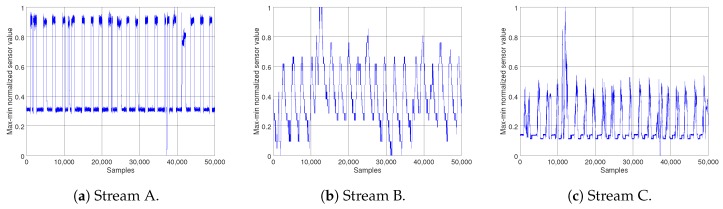
The original sensor data streams. The sampling rate varies for each of the data streams; (**a**) Stream A 100 ms, (**b**) Stream B 500 ms, and (**c**) Stream C 200 ms.

The reduced number of packet transmissions was measured and evaluated using a mathematical analysis of our model implemented in Matlab. We used three different data streams, collected from sensor devices monitoring various aspects of an industrial environment. All of the data are noisy, and each of them have different sampling rates and show diverse behaviors, see Figure 4. To describe average model-performance, the difference between the simulated data stream at the fog node and the original data stream was measured by Mean Square Error (MSE); this is when the fog node was only provided with the statistical information received form the sensor device. Furthermore, we compared the performance of the proposed model to a moving average model, by comparing their relative errors on simulating a data stream and similar parameters setting.

The total end-to-end delay was evaluated and measured in our testbed system, measuring from sensor node to end user application, running either towards the cloud or the fog system. This delay was measured in three steps. First between the sensor mote to the fog node, secondly between the fog node and the cloud node, and lastly between the cloud and end user application. The total delay time can also be represented by Equation (17), where $d_{total}$ is the total delay, $d_{sensor}$ is the sensor delay, $d_{serial}$ is the delay of the reading of the serial communication from the mote, $d_{fog}$ is the fog delay for sending MQTT messages, and $d_{coud}$ is the cloud REST interface delay.

(17) $$d_{total}=d_{sensor}+d_{serial}+d_{fog}+d_{cloud}$$

The sensor delay was measured on the TelosB motes. The end-to-end delay at the sensor network layer can be measured by considering four elements: transmission delay, propagation delay, processing delay and queuing delay. To compare the effect of added computational overhead at sensor level, we compared the end-to-end delay of a unicast process with the delay of the proposed model. The payload size was the same in both settings. The delay was measured and the performance was evaluated using simulation scenarios, with the same setting as the implementation on TelosB motes, defined in Cooja [28]. The fog to cloud communication was measured on the fog devices with the Java program that published sensor values to the cloud, and finally the cloud system was evaluated using an end user Java application accessing the REST interface of the cloud. The query time of both the fog and cloud systems were also evaluated in the testbed. To measure this we used the measurement laptop running a Java program that performs and measures the query response times of the respective REST interfaces. Finally, we evaluated and measured the scalability of the fog nodes. Since we expect each fog device to handle multiple sensor motes and their models, we had to determine the maximum amount of sensor values that our fog device (Raspberry Pi) could handle. All these measurements were made on the local network, which means that they add up to an indication of what the best case minimum response time of the system can be.

## 6. Results and Discussion

We present and evaluate the results of the model and the testbed separately. The model results present measurements and evaluations of the sensor model itself, and its effectiveness in terms of reduced number of packet transmissions and reproducibility of the approximated sensor values based on the model parameters at the fog node. The testbed results present the features and measurements on how well the sensor network layer, fog computing layer and cloud computing layer collaborate and perform as one system. The implications of these results are discussed at the end of this section.

### 6.1. Sensor Data Experiments

The average model performance and the reduced number of packet transmissions were measured and evaluated by implementing the proposed model in Matlab. Forty thousand continuous data points from each of the data streams were sampled.

**Figure 5:**
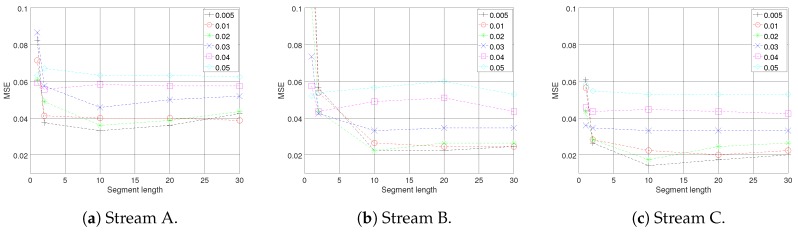
The model MSE measure comparison with respect to the segment length and the trend threshold.

According to the model explained in Section 3, two parameters of trend threshold θ and maximum number of data points in each segment, as well as segment length *m*, have the highest effect on the model performance. The reason is that they indicate the level of trade off between energy efficiency, with respect to transmission reduction, and sensitivity of the model in trend and change detection, which has a direct effect on the accuracy of the simulated data stream at the fog node. Several experiments with various values of θ and *m* for each of the data streams were conducted, to investigate these effects on simulation accuracy at the fog node in terms of mean square error, and to choose the most appropriate set of parameters for the model. Figure 5 presents the results. As can be seen from the graphs, the mean error of the simulated data streams at the fog node has the sharpest decline for all sets of parameters, when the simulations were based on statistical information from two data points, m=2. From this point on the results of each of the data streams show different patterns; nonetheless, the best parameter set for all of the data streams, with respect to the MSE, was achieved by {θ=0.005,m=10}, see Figure 5a–c. At this point for almost all sets of parameters, the trend reversed and has raised steadily. The complete results are summarized in Table 1.

**Figure 6:**
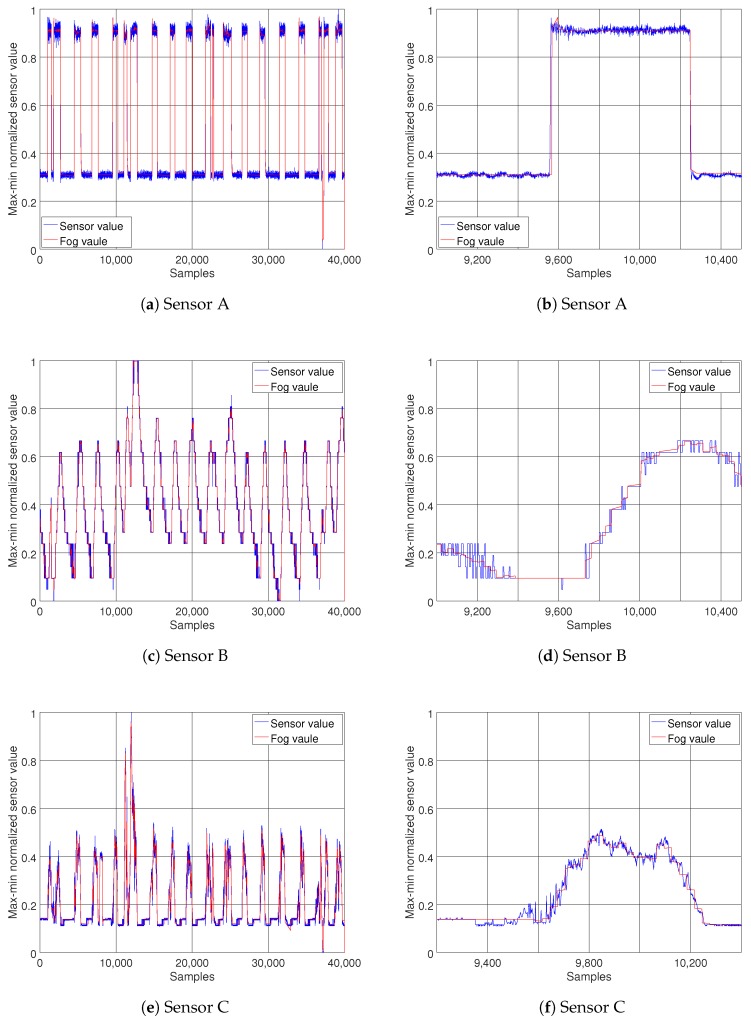
Comparisons between normalized sensor data (blue) and the simulated sensor stream (red). The left column (**a**,**c**,**e**), shows the performance of the proposed method for the duration of the experiment (40,000 samples) on different streams. To illustrate the results in more detail, the right column (**b**,**d**,**f**), shows a zoomed in view of a shorter interval (1200 samples on the same streams).

It should be noted that, when choosing the maximum length of the segment, although a greater *m* means fewer transmissions, hence achieving more energy efficiency, it also means more delay on detecting trend changes and state switches. It also indicates decreased accuracy, since the summary information would be an approximation of a larger number of data points. So although smaller values of *m* might seem encouraging, for improving the energy efficiency, it is better for them to be excluded to prevent accuracy loss. Looking at Figure 5, it can be seen that the MSE shows a fairly stable trend when segment length increases from 10 to 20, with θ=0.02 for stream A and θ=0.01 for streams B and C. For this reason, the parameter set {θ=0.01,m=20} for streams B and C, and {θ=0.02,m=20} for A, were chosen to test the performance of the proposed model. After setting the model parameters, several experiments were carried out for all three data streams. The red lines on Figure 6 show the simulated data streams at the fog node, and the blue lines illustrate the original data streams. On the left column of Figure 6a,c,e, the results of the model for the duration of the experiment are presented. To illustrate the results in more detail, Figure 6b,d,f show zoomed in versions of the same data streams. It is clearly observable that the model captures the behavior of the data streams with good accuracy and low delay.

For this experiment, 40,000 data points from each of the data streams were used. The number of packets sent to a fog node after deploying the model reduced to 812, 898 and 926 for A, B and C, respectively; that is a communication reduction ratio of 49 to 1, 45 to 1 and 43 to 1. In total, for all the data streams, only 2636 of the data points (from the original 120,000 data points) were transmitted. Hence, approximately 2.2% of the packets were sent from sensor devices to a fog node. To compare the performance of the proposed method, the MSE measure of the base model was obtained, with respect to the segment length, see Table 2. As mentioned before, the best parameters set for the proposed method was {θ=0.005,m=10}, with respect to MSE of the simulated data streams at fog node; in addition, the MSE measure was fairly stable with segment length m=10 to m=20. Choosing the parameter set {θ=0.005,m=20}, we can compare the simulation accuracy of the two models. The MSE measure of the data streams A, B, and C were 0.036, 0.022, and 0.017, respectively, when the proposed model was used to simulate the data streams at the fog node; these values for the base model were A = 0.43, B = 0.025, and C = 0.019. It is clear that the proposed method was able to simulate the data stream with a higher accuracy. In terms of transmission reduction, the proposed model simulated the data streams A, B and C with MSE of 0.038, 0.024 and 0.02, respectively, when transmitting one packet every 20 data points, with the most appropriate parameter set. For the base model to achieve the same level of accuracy, the maximum segment length cannot be more than 17 for stream A, 19 for stream B, and 23 for stream C. This means that approximately 15.5% of all the packets need to be sent, which makes the communication cost of the base model 7.5 times more than the proposed model.

Considering the presented results, it is reasonable to conclude that the proposed model can successfully reduce the number of packet transmissions, while keeping the error of the simulated data stream, using only statistical information, within an acceptable range.

### 6.2. Testbed Evaluation

**Figure 7:**
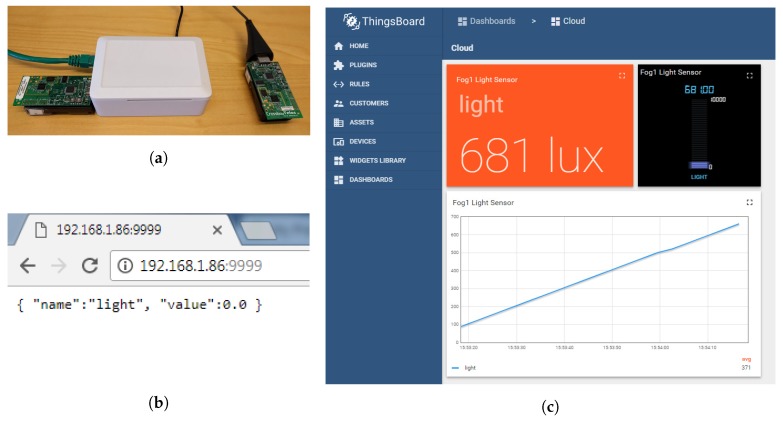
The testbed experimental setup: (**a**) Sensor motes and fog node with attached sensor gateway; (**b**) REST interface, running on fog node, in a regular web browser; (**c**) Cloud dashboard with regenerated sensor values by the proposed model.

The proposed cloud, fog and sensor system works as expected. The sensor mote generates a sensor model from the raw sensor values, which is sent to the sensor gateway to reduce the required wireless communication. The fog system retrieves the model parameters from the sensor network layer, and regenerates the sensor values from the model parameters. The fog system both provides the sensor values directly to the end-user applications via a REST interface and sends the values to the cloud system via the MQTT protocol. On the cloud system, the values are persistently stored and the end user applications can access the sensor values in a more user-friendly manner. Figure 7a shows the sensor motes and fog node with attached sensor gateway. A screenshot of the cloud dashboard with sensor values from the sensor motes via the model and fog device can be seen in Figure 7c. The figure shows three different views of the same sensor value, as a card with exact value, as a digital gauge, and as an animated graph. Figure 7b shows the output from the REST interface running on the fog devices. The output is a simple JSON object with the sensor name and its value.

As previously stated, the end-to-end delay was evaluated and measured in the testbed system. It was measured by adding together each of the steps in the proposed system. Firstly, from sensor node to end user application, secondly between the sensor mote to the fog node, thirdly between the fog node and the cloud node, and lastly between the cloud and end user application.

Each measurement was made 1000 times and the results of the evaluations can be seen in Table 3, where μ denotes the mean and σ denotes the standard deviation of the measurements. Hence, we can see that the total delay is on average 180 ms with a standard deviation of 37 ms. It is, however, important to note that this is made in an optimal network situation where all devices are on the same network.

The query time of both the fog and cloud systems were also evaluated in the testbed. To measure query time we used the measurement laptop running a Java program that performs and measures the query response times of the respective REST interfaces on the fog and cloud. Results can be seen in Table 4.

The experiments on the sensor layer, on both hardware implementation and Cooja simulation, show that the computational overhead has a negligible effect on delay with the chosen precision in the testbed, which is in the order of milliseconds, and the process still fits within the required sampling rate of 1 sample per second. The computational cost adds to processing time within the allocated time slot in magnitude of less than 2 ms. Finally, the scalability of the fog nodes was evaluated. Since we expect each fog device to handle multiple sensor motes and their models, we had to determine the maximum number of sensor values the fog device (Raspberry Pi) could handle from each sensor. According to measurement, the Raspberry Pi used on average 3.4 ms with a standard deviation of 1.8 ms for the serial communication to handle each sensor model update, which translates to about 290 sensor values per second. This means that each fog node can scale up to 290 sensor model updates per second. The realization of the proposed model in the form of the testbed system shows acceptable performance. Considering the presented results it is conceivable that the proposed model can meet the required latency of monitoring systems in industrial scenarios, as it can keep the performance above the required accuracy threshold.

### 6.3. Discussion

Considering the presented results, the distributed modeling at the WSN layer implemented on sensor devices, clearly reduces communication, hence saving spectrum. The simulation results of the fog layer clearly shows how approximations of the model parameter can regenerate the data stream at the fog node with acceptable accuracy. This is possible by providing the sensor nodes with transmission opportunities when detecting change points, instead of limiting them to a scheduled-based packet transmission. Furthermore, the prediction accuracy is enhanced by using more relevant statistical information about data streams. In addition to the presented results, an advantage of the proposed approach in comparison to other studies in this field, is that the model reduces some of the negative effects of wireless sensor communication on the performance of the learning algorithm, namely, synchronization of motes and high rate of dropped packets that adds additional delays to the learning process, and introduces the missing values problem to the learning algorithm.

It is worth mentioning that the presented model and testbed system are still under development. We consider the presented study a preliminary investigation of benefits of edge computing and its possible contribution to enhance network performance in IIoT. Moreover, we showed that it is possible to design cross-layer frameworks and to develop decentralized system models to implement collaborative systems by facilitating different communication standards. In the presented work, there are aspects that deserve more in-depth investigation. In the WSN layer, the complexity of the model can be reduced to make it more suitable for resource constraint devices. In addition, incorporating a more sophisticated medium access control protocol with prioritized packet handling mechanisms can be used to improve the reliability of the proposed model and expand its applicability over scenarios with various characteristics. Since the fog layer provides a localized view over the network, a fault detection mechanism can be deployed as a step toward decentralized fault detection and possible performance prediction. These are topics for future work.

## 7. Conclusions

The goal of this research was to answer the three research questions in Section 1. The first question involved investigating to what extent we can save energy and reduce the number of packet transmissions from sensor devices by introducing a data stream model learned at the WSN layer and approximated at the fog layer. We found that by deploying the proposed model, the number of packets sent over the wireless link can be reduced by 98%, with negligible accuracy loss. The second research question was to investigate if this model can be practically implemented and utilized in a fog computing layer. This was shown in an operational testbed system with real-world hardware. Using TelosB motes as sensor devices, Raspberry Pi devices as fog nodes, and ThingsBoard as an IoT cloud. The final research question aimed at investigating the expected end-to-end delay and information query times of such a system, and find out whether it can be used in industrial monitoring scenarios. To test this, measurements were made on the testbed system. The measurements show that the fog devices can handle 290 sensor model updates per second, and have an average of 180 ms delay from end to end. The measurements also provide an information query time of 5.3 ms for the fog, and 8.9 ms for the cloud under ideal network conditions. Hence, the proposed system shows that a combination of fog and cloud computing including a distributed data stream modeling for wireless sensor networks shows great promise for Industrial Internet of Things applications.

## Figures and Tables

**Table 1:** The mean square error of different settings for segment length (m) and trend threshold (θ).

	Stream A					Stream B					Stream C				
*θ*\m	1	2	10	20	30	1	2	10	20	30	1	2	10	20	30
0.005	0.08	0.03	0.03	0.03	0.04	0.15	0.05	0.02	0.02	0.02	0.06	0.02	0.01	0.01	0.02
0.01	0.07	0.04	0.04	0.04	0.38	0.14	0.05	0.02	0.02	0.02	0.05	0.02	0.02	0.02	0.02
0.02	0.06	0.04	0.03	0.03	0.43	0.10	0.04	0.02	0.02	0.02	0.04	0.02	0.01	0.02	0.02
0.03	0.08	0.05	0.04	0.05	0.05	0.07	0.04	0.03	0.03	0.03	0.03	0.03	0.03	0.03	0.03
0.04	0.05	0.05	0.05	0.05	0.05	0.05	0.04	0.04	0.05	0.04	0.04	0.04	0.04	0.04	0.04
0.05	0.06	0.06	0.06	0.06	0.06	0.05	0.05	0.05	0.06	0.05	0.05	0.05	0.05	0.05	0.05

**Table 2:** The mean square error of sending the average value with different segment length.

m	16	17	18	19	20	21	22	23	24	25	26	27
**Stream A**	0.038	0.039	0.04	0.04	0.043	0.043	0.045	0.046	0.046	0.049	0.05	0.05
**Stream B**	0.024	0.024	0.024	0.024	0.025	0.025	0.025	0.025	0.025	0.025	0.026	0.026
**Stream C**	0.017	0.018	0.018	0.019	0.019	0.02	0.020	0.020	0.021	0.022	0.022	0.023

**Table 3:** Delay measurements split into each step.

Delay Measurement	μ	σ
$d_{sensor}$	140 ms	14 ms
$d_{serial}$	3.4 ms	1.8 ms
$d_{fog}$	32 ms	34 ms
$d_{coud}$	8.9 ms	7.1 ms
$d_{total}$	180 ms	37 ms

**Table 4:** Query time of the REST interfaces.

Query Measurement	μ	σ
**Fog**	5.3 ms	9.0 ms
**Cloud**	8.9 ms	7.1 ms

## References

[1] Atzori L., Iera A., Morabito G. (2010). The internet of things: A survey. Comput. Netw..

[2] Da Xu L., He W., Li S. (2014). Internet of things in industries: A survey. IEEE Trans. Ind. Inform..

[3] Bonomi F., Milito R., Zhu J., Addepalli S. Fog computing and its role in the internet of things. Proceedings of the First Edition of the MCC Workshop on Mobile Cloud Computing.

[4] Han G., Dong Y., Guo H., Shu L., Wu D. (2015). Cross-layer optimized routing in wireless sensor networks with duty cycle and energy harvesting. Wirel. Commun. Mob. Comput..

[5] Rasouli H., Kavian Y.S., Rashvand H.F. (2014). ADCA: Adaptive duty cycle algorithm for energy efficient IEEE 802.15.4 beacon-enabled wireless sensor networks. IEEE Sens. J..

[6] Xie R., Liu A., Gao J. (2016). A residual energy aware schedule scheme for WSNs employing adjustable awake/sleep duty cycle. Wirel. Pers. Commun..

[7] Choudhury N., Matam R., Mukherjee M., Shu L. Adaptive Duty Cycling in IEEE 802.15. 4 Cluster Tree Networks Using MAC Parameters. Proceedings of the 18th ACM International Symposium on Mobile Ad Hoc Networking and Computing.

[8] Huang M., Liu A., Wang T., Huang C. (2018). Green data gathering under delay differentiated services constraint for internet of things. Wirel. Commun. Mob. Comput..

[9] Azharuddin M., Kuila P., Jana P.K. (2015). Energy efficient fault tolerant clustering and routing algorithms for wireless sensor networks. Comput. Electr. Eng..

[10] Naranjo P.G.V., Shojafar M., Mostafaei H., Pooranian Z., Baccarelli E. (2017). P-SEP: A prolong stable election routing algorithm for energy-limited heterogeneous fog-supported wireless sensor networks. J. Supercomput..

[11] Oller J., Demirkol I., Casademont J., Paradells J., Gamm G.U., Reindl L. (2016). Has time come to switch from duty-cycled MAC protocols to wake-up radio for wireless sensor networks?. IEEE/ACM Trans. Netw..

[12] Mahmood A., Shi K., Khatoon S., Xiao M. (2013). Data mining techniques for wireless sensor networks: A survey. Int. J. Distrib. Sens. Netw..

[13] Alsheikh M.A., Lin S., Niyato D., Tan H.P. (2014). Machine learning in wireless sensor networks: Algorithms, strategies, and applications. IEEE Commun. Surv. Tutor..

[14] Guestrin C., Bodik P., Thibaux R., Paskin M., Madden S. Distributed regression: an efficient framework for modeling sensor network data. Proceedings of the 3rd International Symposium on Information Processing in Sensor Networks.

[15] Gelb A. (1974). Applied Optimal Estimation.

[16] Abbeel P., Coates A., Montemerlo M., Ng A.Y., Thrun S. Discriminative Training of Kalman Filters. Proceedings of the Robotics: Science and Systems.

[17] Liu Q., Wang Z., He X., Zhou D. (2015). Event-based recursive distributed filtering over wireless sensor networks. IEEE Trans. Autom. Control.

[18] Leskovec J., Rajaraman A., Ullman J.D. (2014). Mining of Massive Datasets.

[19] Schütz N., Holschneider M. (2011). Detection of trend changes in time series using Bayesian inference. Phys. Rev. E.

[20] Chung F.L., Fu T.C., Ng V., Luk R.W. (2004). An evolutionary approach to pattern-based time series segmentation. IEEE Trans. Evolut. Comput..

[21] Fuchs E., Gruber T., Nitschke J., Sick B. (2010). Online segmentation of time series based on polynomial least-squares approximations. IEEE Trans. Pattern Anal. Mach. Intell..

[22] Wang K., Wang Y., Sun Y., Guo S., Wu J. (2016). Green industrial internet of things architecture: An energy-efficient perspective. IEEE Commun. Mag..

[23] Li H., Ota K., Dong M. (2018). Learning IoT in Edge: Deep Learning for the Internet of Things with Edge Computing. IEEE Netw..

[24] Dautov R., Distefano S., Bruneo D., Longo F., Merlino G., Puliafito A. Pushing Intelligence to the Edge with a Stream Processing Architecture. Proceedings of the 2017 IEEE International Conference on Internet of Things (iThings) and IEEE Green Computing and Communications (GreenCom) and IEEE Cyber, Physical and Social Computing (CPSCom) and IEEE Smart Data (SmartData).

[25] Dunkels A., Gronvall B., Voigt T. Contiki-a lightweight and flexible operating system for tiny networked sensors. Proceedings of the 2004 29th Annual IEEE International Conference on Local Computer Networks.

[26] Crossbow TelosB Mote Plattform, Datasheet. http://www.willow.co.uk/TelosB_Datasheet.pdf.

[27] Single-Chip 2.4 GHz IEEE 802.15.4 Compliant and ZigBee™ Ready RF Transceiver. http://www.ti.com/product/CC2420.

[28] Osterlind F., Dunkels A., Eriksson J., Finne N., Voigt T. Cross-level sensor network simulation with Cooja. Proceedings of the 2006 31st IEEE Conference on Local Computer Networks.

